# Sera from Rheumatoid Arthritis Patients Induce Oxidative Stress and Pro-Angiogenic and Profibrotic Phenotypes in Human Endothelial Cells

**DOI:** 10.3390/jcm13195913

**Published:** 2024-10-03

**Authors:** Roberta Giordo, Anna Maria Posadino, Paola Maccioccu, Giampiero Capobianco, Angelo Zinellu, Gian Luca Erre, Gianfranco Pintus

**Affiliations:** 1 Department of Biomedical Sciences, University of Sassari, 07100 Sassari, Italy; rgiordo@uniss.it (R.G.); posadino@uniss.it (A.M.P.); p.maccioccu@phd.uniss.it (P.M.); azinellu@uniss.it (A.Z.); 2 Gynecologic and Obstetric Clinic, Department of Medicine, Surgery and Pharmacy, University of Sassari, 07100 Sassari, Italy; capobia@uniss.it; 3 Rheumatology Unit, Department of Medicine, Surgery and Pharmacy, University Hospital (AOUSS), University of Sassari, 07100 Sassari, Italy

**Keywords:** rheumatoid arthritis, oxidative stress, angiogenesis, fibrosis, collagen synthesis

## Abstract

**Background:** Rheumatoid arthritis (RA) is a long-term autoimmune condition marked by persistent inflammation of the joints and various systemic complications, including endothelial dysfunction, atherosclerosis, and pulmonary fibrosis. Oxidative stress is a key contributor to the pathogenesis of RA, potentially exacerbating vascular damage and promoting pro-angiogenic and profibrotic processes. **Objective:** This study aims to investigate the effects of sera from RA patients on human umbilical vein endothelial cells (HUVECs), focusing on the induction of oxidative stress, endothelial cell proliferation, migration, and collagen type I synthesis. **Methods:** Twenty-eight serum samples were collected from RA patients and healthy donors (HDs). HUVECs were exposed to these sera, and intracellular reactive oxygen species (ROS) levels were fluorescently detected using H2DCF-DA. Cell viability was assessed using the 3-(4,5-dimethylthiazol-2-yl)-2,5-diphenyltetrazolium bromide (MTT) assay. Cell migration was evaluated through a scratch wound assay, and collagen type I synthesis was measured using a lentiviral vector expressing the green fluorescent protein (GFP) under the control of the human COL1A1 gene promoter. **Results:** Exposure to RA sera resulted in a significant increase in intracellular ROS levels in HUVECs compared to HD sera, indicating an elevated state of oxidative stress. RA sera also promoted endothelial cell proliferation and migration, suggesting a pro-angiogenic stimulus. Additionally, RA sera significantly increased collagen type I synthesis in HUVECs, implicating a potential role in profibrotic processes associated with RA. **Conclusion:** The results of this study emphasize the importance of circulating factors in RA sera in promoting oxidative stress, endothelial dysfunction, and pro-angiogenic and profibrotic phenotypes in endothelial cells. These processes may contribute to the vascular and fibrotic complications observed in RA, highlighting the necessity for additional research into focused therapeutic approaches to alleviate these effects.

## 1. Introduction

Rheumatoid arthritis (RA) is a long-term autoimmune pathological state that predominantly influences the synovium, the lining of the joints, resulting in joint swelling, stiffness, and a progressive loss of joint flexibility [1,2]. The worldwide occurrence of RA is estimated to be approximately 209 cases per 100,000 people, with women being affected at twice the rate of men. Geographic variations in RA prevalence may reflect differences in genetic and environmental factors, such as smoking, viral infections, exposure to air pollution, and variations in the gastrointestinal microbiome [3,4,5].

Along with joint inflammation, RA patients may experience extra-articular manifestations and complications, including osteoporosis, rheumatoid nodules, and disorders affecting the pulmonary, cardiovascular, gastrointestinal, neurological, hematological, and renal systems [6]. Epidemiological studies have shown that RA patients have a 1.5 to 2.0 times higher risk of developing atherosclerosis and other cardiovascular conditions than the general population, likely due to the chronic and systemic inflammatory nature of the disease [7,8]. In RA, the loss of endothelial integrity and function, known as endothelial dysfunction, is crucial in accelerating the development of atherosclerosis and cardiovascular complications [9]. Both large blood vessels and the microvasculature are prone to endothelial dysfunction in RA. While macrovascular endothelial dysfunction is often seen as the primary contributor to atherogenesis, emerging research indicates that microvascular endothelial dysfunction may serve as an early indicator of atherosclerosis and cardiovascular risk [9].

Furthermore, recent findings indicate that serum concentrations of Ischemia-Modified Albumin (IMA) and asymmetric dimethylarginine, molecules that are strictly associated with inflammatory states, ischemia, and oxidative stress, are higher in RA patients compared to the general population [10,11,12]. These elevated levels are significantly correlated with endothelial dysfunction and high disease activity [13]. The excessive production of ROS, caused by an imbalance between the oxidant and antioxidant systems, is a key factor in triggering endothelial dysfunction. Oxidative stress can exacerbate the proinflammatory, pro-thrombotic, and proliferative state of endothelial cells, decrease the formation and accessibility of nitric oxide (NO), and impair vasodilation, barrier function, and angiogenesis [9,14,15]. In this pathological context, endothelial cells may, in turn, increase ROS generation, establishing a positive feedback loop that amplifies both oxidative stress and endothelial damage [16,17].

Further evidence highlights the pivotal role of oxidative stress in the development of RA [18,19], with an excess of ROS and elevated levels of protein oxidation, lipid peroxidation, and DNA damage observed alongside lowered antioxidant defenses in both the synovial fluid and serum of RA patients [18,20,21]. This redox imbalance contributes to cellular damage and tissue deterioration, ultimately affecting major organs [18,22]. Oxidative stress is also linked to increased synovial inflammation and angiogenesis [23]. The hypoxia and tissue hypoperfusion resulting from chronic oxidative damage and inflammation in the synovial microenvironment stimulate angiogenesis to meet the heightened demand for oxygen and nutrients in the hypertrophic joint [24,25].

It is hypothesized that blood-contained pro-oxidant factors may contribute to RA’s pathogenesis by inducing activation and phenotypic changes in human umbilical vein endothelial cells (HUVECs). This study seeks to examine the impacts of serum from RA patients on HUVECs, comparing these effects to those observed with serum from healthy donors (HDs). Specifically, the study will evaluate phenomena related to endothelial dysfunction, including ROS production, cell proliferation and migration, and type 1 collagen synthesis.

## 2. Materials and Methods

### 2.1. Patients 

Twenty serum samples were collected from RA patients’ groups at the Unit of Complex Rheumatology, University of Sassari, Sassari, Italy. All RA patients met the classification criteria established by the American College of Rheumatology and the European League Against Rheumatism [26]. RA patients were participants in the Bio-RA study, which analyzes endothelial dysfunction in RA patients as part of the evaluation of coronary heart disease risk estimation (ClinicalTrials.gov: NCT02341066). Table 1 summarizes the clinical and serological characteristics of the individuals enrolled in the present study.

All participants underwent a thorough assessment and evaluation of traditional cardiovascular risk factors, including smoking status, hypertension, diabetes, and dyslipidemia. In addition, data on positivity for rheumatoid factor (RF), anticitrullinated cyclic peptide antibodies (ACPAs), and inflammation laboratory parameters, such as the erythrocyte sedimentation rate (ESR), were collected. Data were also collected on treatment with disease-modifying antirheumatic drugs (DMARDs); methotrexate (MTX); tumor necrosis factor inhibitors (TNFis); steroids use; and disease activity score-28 (DAS-28), which provides a quantitative measure of disease activity by evaluating 28 specific joints in the body, focusing on those most commonly affected by RA, such as the wrists, elbows, knees, shoulders, and small joints of the hands [27]. Finally, the endothelial function index, Ln-RHI (log-transformed reactive hyperemia index), and the oxidative stress marker, malondialdehyde (MDA), were measured [13,19].

Healthy donors (HDs) were enrolled in this study after completing a screening questionnaire designed to exclude the presence of any underlying vascular or autoimmune diseases. Although we tried to match the HDs for gender, race, and smoking status, male RA patients were more prevalent compared to male controls, and female controls were more prevalent compared to males within the same group (Table 1). The research involving human subjects was conducted in accordance with all relevant national regulations, institutional policies, and the principles outlined in the Helsinki Declaration. The Ethics Committee of Azienda ASL 1 of Sassari (Italy) approved this study (2126/CE-2015 and 2219/CE-2015). All participants included in this study gave their informed consent.

### 2.2. Cell Culture and Treatment

Human umbilical vein endothelial cells (HUVECs) were obtained from PromoCell (Heidelberg, Germany) as described in previous studies [28]. Briefly, the cells were cultured in endothelial cell basal medium, supplemented with the Endothelial Cell Growth Medium MV Supplement Mix (#C-39225) according to the manufacturer’s instructions. Upon reaching confluence, HUVECs were routinely divided and experimentally processed between passages one and four.

Unless otherwise specified, the cells were cultured in 96-well plates (Corning, Lowell, MA, USA) and then experimentally processed in a standard medium supplemented with 5% serum (*v*/*v*) obtained from the study participants. The sera from various subjects were standardized according to their protein contents [29].

### 2.3. Determination of Cell Viability

Cell viability was evaluated using a colorimetric assay based on 3-(4,5-dimethylthiazol-2-yl)-2,5-diphenyltetrazolium bromide (MTT) (Promega, Madison, WI, USA) in 96-well plates (BD Falcon) [30,31]. The MTT reagent (yellow) was taken up by cells and transported to the mitochondria, where it was cleaved by mitochondrial dehydrogenases in viable cells, resulting in the formation of hydrophobic formazan crystals (purple). As this reduction took place exclusively in active mitochondria, the conversion of MTT to purple formazan was directly proportional to the number of proliferating cells.

The purple crystals of formazan created by viable cells were subsequently solubilized in acidified isopropanol and read spectrophotometrically at 570 nm. The amount of formazan produced served as an indicator of the viable cell count. After 24 and 48 h of cell treatment, 20 µL of MTT solution (2 mg/mL) was added to the cell medium, followed by a 2 h incubation at 37 °C. Following incubation, the medium was discarded, and the precipitated formazan was dissolved using 0.04 N HCl solution of isopropanol. The solubilized product was measured at an absorbance of 570 nm using a GENios plus microplate reader (Tecan). The results are reported in absorbance units (ABSs) as the mean ± standard deviation (SD).

### 2.4. Determination of Intracellular ROS

The levels of intracellular ROS were quantified utilizing the ROS-sensitive molecular probe 2’,7’-dichlorodihydrofluorescein diacetate (H2DCF-DA) (Molecular Probes, Eugene, OR, USA) following a previously established method with minor modifications [32,33]. Upon oxidation by intracellular ROS, H2DCF is converted into DCF, a compound whose fluorescence intensity directly indicates the ROS levels inside the cell. Cells were subjected to treatments as outlined in the figure legends, after which they were processed for ROS evaluation.

For the assessment of ROS, the cultured cells were pre-incubated for 30 min in Hank’s Balanced Salt Solution (HBSS) supplemented with 10 µM H2DCF-DA, followed by two washes with HBSS. Working under light-protected conditions, a GENios plus microplate reader (Tecan, Männedorf, Switzerland) was used for fluorescence quantification. The employed excitation and emission wavelengths used were 485 nm and 535 nm, respectively. Variations in fluorescence induced by treatment were monitored kinetically over a 4 h time course. All fluorescence determinations were corrected for the fluorescence of the background and normalized according to the protein content, with results expressed in relative fluorescence unit (RFU) values as the mean ± SD.

### 2.5. Collagen Type I Synthesis Determination

Collagen type I (COL1) synthesis was assessed using the COL1A1-LV-tGFP lentiviral vector, which expresses green fluorescent protein (GFP) driven by human COL1A1 gene promoter [34]. To normalize for cell transduction efficiency, a red fluorescent protein-based lentiviral vector (EF1α-LV-FP602), harboring the red fluorescent protein (FP602) driven by the constitutive elongation factor 1-alpha (EF1α) promoter, was employed [35]. The generation of the stable HUVEC/pCol1GFP-pEFα-FP602 cell line, which contains both pCOL1A1-tGFP and EF1α-FP602 constructs, was performed as previously reported, with minor modifications [36,37].

In brief, HUVECs at 50-60% confluence were infected with the lentiviral particles utilizing lentiviral particles at an optimized multiplicity of infection (MOI) to achieve an infection efficiency of approximately 95%. The stable transduced HUVECs enabled the simultaneous kinetic assessment of several samples in a 96-well plate using relatively a small volume of subject serum [34]. HUVEC/pCol1GFP-pEFα-FP602 cells were grown in standard medium supplemented with 5% (*v*/*v*) serum from RA patients and 5% serum from healthy donors (HDs). The treatment-elicited changes in GFP fluorescence were monitored kinetically over a 4 h period using the microplate reader Tecan GENios Plus (Tecan, Mannedorf, Switzerland).

For fluorescence determination, excitation wavelengths of 485 nm for GFP and 535 nm for FP602 were used, with corresponding emission wavelengths of 535 nm and 590 nm, respectively. Data were normalized for transduction efficiency by calculating the ratio of pCOL1A1-LV-tGFP to EF1α-LV-FP602 fluorescence and are presented as the mean ± SD of relative fluorescence unit (RFU) values.

### 2.6. Cell Migration

Cell migration was evaluated using a scratch wound assay [38]. HUVECs were seeded in 6-well plates and allowed to grow until they reached 80–90% confluence. Once the cells reached confluence, a straight-line scratch was created in the cell layer using a 1 mL pipette tip. The detached cells were then washed away, and the remaining cells were cultured for an additional 48 h in basal medium supplemented with 5% (*v*/*v*) serum from RA patients or 5% (*v*/*v*) serum from healthy donors (HDs).

Cell migration across the scratch was monitored at 24 and 48 h post-scratch. Images of the scratch wounds were captured at 0, 24, and 48 h after the scratch was made, using an inverted light microscope (Zeiss Axiovert 200M) equipped with a Zeiss AxioCam MRm camera (Carl Zeiss Microscopy, Jena, Germany). Wound edge distance was quantified using ImageJ software, version 1.53t (National Institutes of Health, Bethesda, MD, USA).

### 2.7. Statistical Analysis

The statistical differences between two groups were analyzed by unpaired *t*-test with Welch’s correction. In contrast, statistical differences among different groups were analyzed by two-way analysis of variance, followed by Tukey’s post hoc test for multiple comparisons. All statistical analyses were conducted using GraphPad Prism version 9.00 for Windows (GraphPad Software, San Diego, CA, USA), with *p*-values < 0.05 considered statistically significant.

## 3. Results and Discussion

RA patients enrolled in this study comprised 53.54% males and 46.43% females, with an average age of 61.05 ± 15.31 years (Table 1). The study evaluated various drug therapies (including steroids, NSAIDs, methotrexate, DMARDs, and TNFi) across all subjects, alongside assessments of endothelial damage—measured by a low Ln-RHI index—and oxidative stress, quantified by a by-product of lipid peroxidation, the levels of malondialdehyde (MDA) (Table 1). Current evidence highlights oxidative stress as a key contributor to RA’s pathogenesis [18,39], strongly linking it to vascular endothelial damage, the primary cause of accelerated atherogenesis and cardiovascular complications [39,40,41].

**Figure 1:**
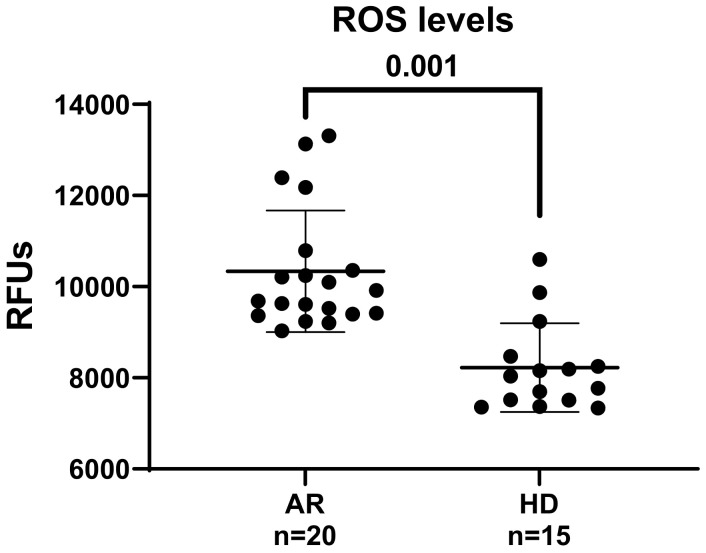
Effect of RA sera on intracellular ros levels in human umbilical vein endothelial cells (HUVECs). To investigate the effect of sera from rheumatoid arthritis (RA) patients on intracellular reactive oxygen species (ROS) levels, sub-confluent HUVECs were pre-loaded with 10 μM of H2-DCFDA, a fluorescent probe for ROS detection. The cells were then grown in a basal medium supplemented with 5% (*v*/*v*) sera from either RA patients or healthy donors (HDs). The ROS levels inside the cells were measured kinetically over a 4 h period, with the data from the first hour used for comparative analysis. Fluorescence intensity data, indicative of intracellular ROS levels, were normalized to protein content and expressed as relative fluorescence units (RFUs). The results are presented as mean values, with standard deviations indicated by horizontal lines. A Welch’s correction-modified unpaired *t*-test was employed to analyze the statistical differences among the studied groups, considering *p*-values lower than 0.05 statistically significant.

Additionally, excess ROS, increased levels of protein oxidation, lipid peroxidation, DNA damage, and lowered antioxidant defenses have been observed in both the synovial fluid and serum of RA patients [20,21,42,43]. To further investigate this aspect, we first examined whether exposing HUVECs to RA patients’ sera could induce a rise in intracellular ROS levels, leading to oxidative stress. For ROS detection, sub-confluent HUVECs were added with 10 µM H_2_DCFDA and growth in basal medium containing 5% (*v*/*v*) sera from healthy donors (HDs) and RA patients. The levels of intracellular ROS were kinetically measured over a 4 h time course, with values at 1 h (steady state) used for comparison (Figure 1).

As shown in Figure 1, the levels of intracellular ROS in HUVECs treated with RA sera were significantly elevated compared to cells exposed to HD sera, indicating that RA patients possess circulating factors that enhance the oxidative state of the endothelium. The findings suggest that immune response mechanisms, which are at least partially independent of disease activity and patient-reported outcomes, may contribute to pro-angiogenic and profibrotic responses in HUVECs. The elevated ROS levels and hypoxic conditions contribute to chronic oxidative damage in the synovial microenvironment of RA, amplifying inflammatory responses and pro-angiogenic stimuli to meet the hypertrophic joint’s oxygen and nutrient demands [23,24,44].

**Figure 2:**
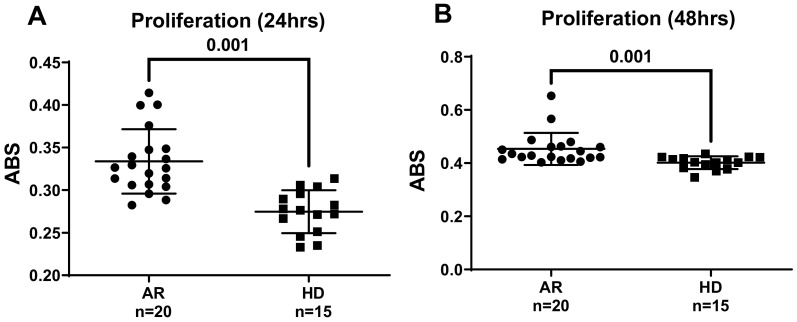
(**A**,**B**) Impact of RA sera on the proliferation of human umbilical vein endothelial cells (HUVECs). Sub-confluent HUVECs were grown for 24 h (**A**) and 48 h (**B**) in a basal medium supplemented with 5% (*v*/*v*) sera from either rheumatoid arthritis (RA) patients or healthy donors (HDs). After the respective incubation periods, cell viability was assessed according to the protocol described in the Materials and Methods section. The absorbance (ABS) results are presented as mean values, with standard deviations indicated by horizontal lines. A Welch’s correction-modified unpaired *t*-test was employed to analyze the statistical differences among the studied groups, considering *p*-values lower than 0.05 statistically significant.

Vascular endothelial growth factor (VEGF) is a major pro-angiogenic molecule released by endothelial cells under hypoxic conditions [45,46]. VEGF binds to the VEGFR2 receptor, directly stimulating ROS production through NOX activation and initiating signal transduction pathways that promote angiogenic processes, including endothelial cell proliferation, migration, and differentiation [40]. Hypoxia also increases ROS production via HIF-1α activation, further enhancing VEGF secretion and promoting angiogenesis [47]. Conversely, hypoxia triggers an increase in ROS production by activating HIF-1α. This activation subsequently enhances VEGF secretion, ultimately promoting angiogenesis [47]. Consistent with this, elevated VEGF levels are found in RA patients’ serum and synovial fluid [48,49]. In this light, we then investigated whether RA patients’ sera could influence the proliferative and migratory capacity of HUVECs. The HUVECs’ proliferation rate was measured using an MTT assay after 24 and 48 h of treatment with sera from RA patients and healthy donors (HDs). As shown in Figure 2, sera from RA patients induced a phenotypic switch in HUVECs, resulting in increased cell proliferation after both 24 h (Figure 2A) and 48 h (Figure 2B) of exposure.

Cell migration is a fundamental biological process and essential for various physiological and pathological events involving the movement of cells in response to specific signals [50]. To assess whether RA sera could alter HUVEC migration, a wound healing migration assay was performed.

**Figure 3:**
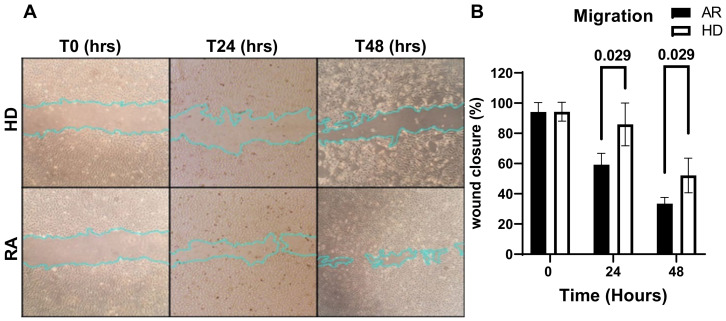
(**A**,**B**) Effect of RA sera on the migration of human umbilical vein endothelial cells (HUVECs). Sub-confluent HUVECs were grown for 24 and 48 h in a basal medium supplemented with 5% (*v*/*v*) sera from rheumatoid arthritis (RA) patients or healthy donors (HDs). Wound-healing assays were conducted at 0, 24, and 48 h to assess cell migration in both RA-treated HUVECs and HD-treated HUVECs, which served as controls. (**A**) Representative phase-contrast microscopy images illustrate the area covered by cells at 0, 24, and 48 h following wounding. Original magnification: 10×. After 24 h, HUVECs treated with RA sera migrated faster than those treated with HD sera, nearly closing the wound gap after 48 h, as indicated by the manually drawn overlays. (**B**) The percentage of wound closure was quantitatively assessed by measuring the wound edges with ImageJ software, version 1.53t. The results are presented as mean values, with standard deviations indicated by horizontal lines. Statistical differences between the groups were assessed using a two-way analysis of variance, followed by Tukey’s post hoc test for multiple comparisons, considering *p*-values < 0.05 statistically significant.

The data reported in Figure 3A,B show a significant increase in the migration rates of HUVECs treated with RA sera at 24 and 48 h compared to those treated with HD sera. Overall, these findings suggest that angiogenic factors that are present in RA patients’ sera may activate HUVECs, leading to a more proliferative and migratory phenotype.

Up to 30% of individuals with RA can develop lung diseases, including pulmonary fibrosis, characterized by progressive structural damage in the lungs due to uncontrolled collagen deposition [51,52,53]. High levels of profibrotic and proinflammatory mediators in the blood and synovial tissue of RA patients can trigger a profibrotic switch, where activated fibroblasts differentiate into myofibroblasts, leading to increased collagen secretion in the interstitial lung tissues [52,54]. Endothelial cells can also contribute to the progression of fibrosis, either by undergoing an endothelial-to-myofibroblast transition (EndMT) or by secreting additional profibrotic and proinflammatory mediators [55,56].

We further examined whether exposure to RA patients’ sera could promote collagen type I (COL1A1) synthesis in our endothelial model. COL1A1 synthesis was determined using the HUVEC/pCol1GFP-pEFα-FP602 cell line, created by co-transducing HUVECs with pEFα-FP602 and pCOL1A1-tGFP lentiviral particles. This method allows for the simultaneous kinetic assessment of several samples in a 96-well plate using a relatively small quantity of serum.

**Figure 4:**
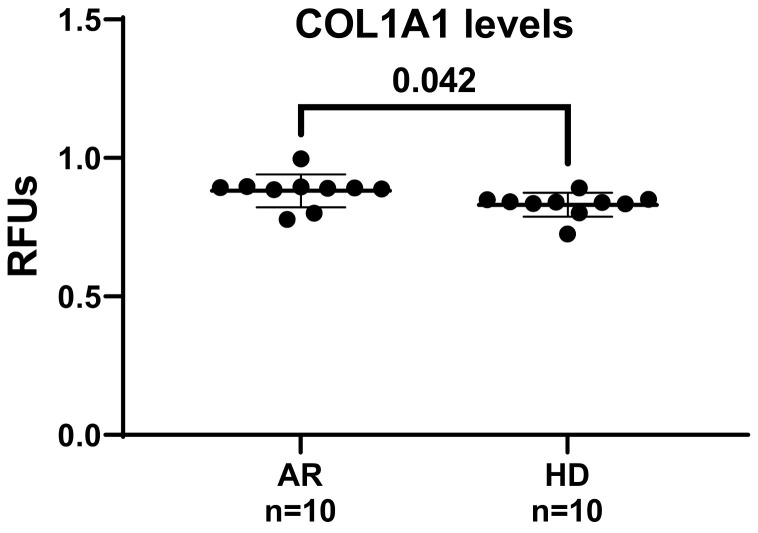
Effects of RA sera on collagen promoter activity in HUVECs. Sub-confluent HUVEC/pCol1GFP-pEFα-FP602 cells were cultured in a standard medium supplemented with 5% (*v*/*v*) sera from rheumatoid arthritis (RA) patients and healthy donors (HDs). The activation of the collagen type I (COL1A1) promoter was monitored kinetically over a 4 h period, with the first hour’s values used for comparative analysis. Data were expressed as relative fluorescence units (RFUs) and corrected for transduction efficiency using the ratio of pCOL1A1-LV-tGFP (green fluorescence) to EF-LV-FP602 (red fluorescence). The results are presented as mean values, with standard deviations indicated by horizontal lines. A Welch’s correction-modified unpaired *t*-test was employed to analyze the statistical differences among the studied groups, considering *p*-values lower than 0.05 statistically significant.

Cells were stimulated with 5% (*v*/*v*) sera from RA patients and HDs, and COL1A1 synthesis was monitored kinetically over a 4 h period. Values at 2 h (steady state) were utilized for comparative analysis (Figure 4). As shown in Figure 4, the exposure of HUVECs to RA sera led to a progressive, time-dependent increase in COL1A1 promoter activity, with significantly elevated levels observed at 2 h in cells treated with RA sera compared to those treated with HD sera. This suggests that profibrotic processes leading to pulmonary fibrosis in RA patients may involve not only changes in connective tissue but also in the blood vessels.

**Figure 5:**
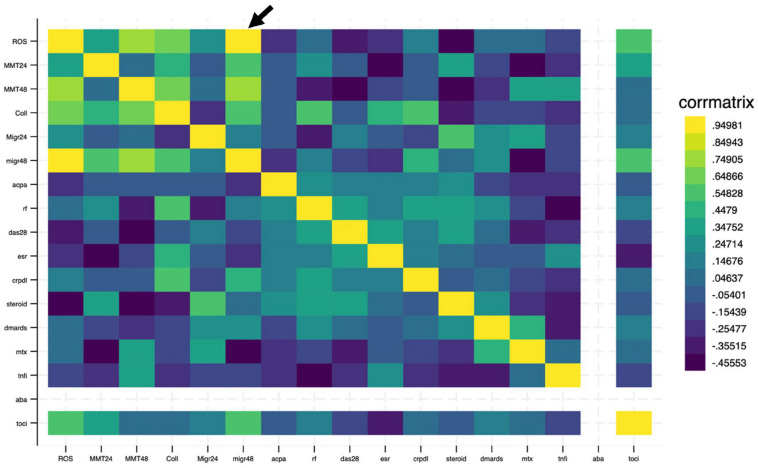
Correlation matrix illustrating the relationships between the clinical parameters of patients and the in vitro experimental data, as well as the correlations among the various in vitro experimental outcomes. The arrow highlights the significant correlation between ROS levels and cell migration at 48 h.

We then performed a set of analyses to explore potential correlations between clinical parameters and experimental outcomes, as well as among different experimental variables such as ROS levels, cell proliferation, and collagen synthesis. As shown in the correlation matrix (Figure 5), no statistically significant correlations were observed between the in vitro experimental results and the clinical features of RA patients.

Regarding the relationships among ROS levels, cell proliferation, and collagen synthesis, we identified a significant positive correlation between ROS levels and cell migration at 48 h, indicating that higher ROS production is associated with enhanced migratory capacity (r = 0.98, *p* < 0.005) (Figure 5). However, no statistically significant correlations were found between ROS and other parameters, such as cell proliferation and collagen activity. The limited sample size in our study likely contributed to the absence of broader significant findings. It is important to note that small sample sizes are commonly used in in vitro and ex vivo studies due to practical limitations, including the availability of biological material and the complexity of experimental protocols [57,58]. While smaller sample sizes may reduce the statistical power, thereby limiting the detection of subtle relationships between variables, they are often necessary in early-stage exploratory research. Future studies should aim to include larger sample sizes to increase statistical power and allow for a more thorough investigation of the interactions between ROS and other cellular processes, such as proliferation and collagen synthesis.

## 4. Future Perspectives

The results of this study open several avenues for future research. Firstly, further investigation is needed to identify the specific circulating factors in RA sera that are responsible for inducing oxidative stress and endothelial dysfunction. Understanding the molecular pathways that are involved in these processes could lead to the development of targeted therapies aimed at mitigating vascular damage in RA patients. Secondly, exploring the role of other cell types, such as immune cells and fibroblasts, in the context of RA-induced endothelial dysfunction could provide a more comprehensive understanding of the disease’s pathogenesis. Additionally, longitudinal studies involving RA patients at different stages of the disease could help clarify the temporal relationship between oxidative stress, angiogenesis, and fibrosis, potentially leading to earlier diagnostic markers and therapeutic interventions. Finally, given the potential link between RA and cardiovascular diseases, it would be valuable to assess whether strategies aimed at reducing oxidative stress and inhibiting pro-angiogenic signals can effectively prevent or slow down the progression of RA-associated cardiovascular and pulmonary complications.

## 5. Limitations

We acknowledge that the experimental research presented in this study has several limitations. Sample Size: The relatively small number of subjects and samples used in the experiments may limit the generalizability of the findings to a broader population. In Vitro Conditions: The use of HUVECs and wound-healing assays in vitro does not fully replicate the complexity of in vivo environments. Consequently, the results observed under laboratory conditions may not entirely reflect cellular behavior within living organisms, limiting the applicability of the findings to clinical settings. Time Constraints for Observations: The observations made at 24 and 48 h may not adequately capture the long-term effects of the experimental treatments. Certain processes, such as fibrosis and cell migration, may have delayed outcomes that are not observable within the observation period reported. Variability of RA Sera: This study employs sera from RA patients, who may have undergone different pharmacological treatments. Additionally, RA manifests heterogeneously across patients, which may result in variability in sera composition. Sex Imbalance: A sex imbalance is present in our study, with more male RA patients compared to male controls and more female controls compared to male controls. However, this imbalance reflects the natural sex distribution seen in RA [59], where females are more commonly affected by the disease. Additionally, the control group composition reflects the availability of healthy volunteers, which often skews toward females in non-interventional studies. While this distribution does not undermine the validity of our findings, we acknowledge that the sex imbalance should be noted as a limitation of this study, as it could influence immune responses and disease progression in RA.

In summary, the aforementioned points may introduce variability in the results, potentially affecting the reproducibility and consistency of the findings. These limitations should be considered when interpreting the results and their potential application to clinical settings.

## 6. Conclusions

This work provides significant insights into the mechanisms underlying vascular damage in RA by focusing on the effects of sera from RA patients on HUVECs. The study results demonstrate that RA sera can significantly increase intracellular ROS levels, induce a pro-angiogenic phenotype, and enhance collagen type I synthesis in endothelial cells. These findings suggest that circulating factors in RA patients’ sera may contribute to endothelial dysfunction by promoting oxidative stress and facilitating pro-angiogenic and profibrotic processes. These processes likely play a crucial role in the progression of RA-associated vascular and fibrotic complications, such as atherosclerosis and pulmonary fibrosis.

## Figures and Tables

**Table 1:** Clinical and serological characteristics of the subjects enrolled in this study.

Variables	RA (n = 20)	HD (n = 15)
Age at serum sampling (years) *	58 ± 7	52 ± 11
Male n (%)	11 (55)	4 (27)
Female n (%)	9 (45)	11 (73)
Smoking status n (%)	14 (70)	6 (40)
ACPA positivity n (%)	10 (50)	
RF positivity n (%)	16 (80)	
DMARD use n (%)	16 (80)	
MTX use n (%)	11 (55)	
TNFi use n (%)	5 (25)	
Steroids use n (%)	10 (50)	
Steroids dose (mg/day) *	4.8 ± 0.8	
ESR *	19.4 ± 12.5	
CRP level mg/dL	0.5 ± 0.5	
Disease duration before sampling (months)	130 ± 91	
DAS-28 *	3.7 ± 1.3	
- remission, n (%) - low disease activity, n (%) - moderate disease activity, n (%) - high disease activity, n (%)	6 (30) 2 (10) 8 (40) 4 (20)	
Ln-RHI *	0.6 ± 0.4	
MDA (µmol/L) *	3.7 ± 0.5	

All values are presented as numbers (%) unless otherwise stated. * Mean ± SD. RA, rheumatoid arthritis; HDs, healthy donors; ACPA, anticitrullinated cyclic peptide antibodies; DMARDs, disease-modifying antirheumatic drugs; RF, rheumatoid factor; ESR, erythrocyte sedimentation rate; CRP, c-reactive protein; MTX, methotrexate; TNFis, tumor necrosis factor inhibitors; Ln-RHI, log-transformed reactive hyperemia index; MDA, malondialdehyde; DAS-28, disease activity score-28.

## Data Availability

All relevant data are available within the manuscript.
